# Semimembranosus Tendon Entrapment by an All-Inside Meniscal Suture

**DOI:** 10.7759/cureus.29746

**Published:** 2022-09-29

**Authors:** Emil Haikal, Jad Mansour, Mohammad Daher, Alfred Khoury

**Affiliations:** 1 Orthopedics, Lebanese American University Medical Center, Beirut, LBN

**Keywords:** entrapment, semimembranosus, meniscal tear, meniscal suture, all-inside

## Abstract

Meniscal suturing has become the gold standard when it comes to meniscal tears in vascularized areas, especially in the younger population. The all-inside meniscal suturing technique has gained popularity in the past year due to decreased operative time as well as decreased risk of adverse events, as compared to other modalities. However, several complications have been reported with the all-inside technique, including soft tissue and neurovascular injury. This is the first case reporting a semimembranosus tendon entrapment following an all-inside medial meniscal suture. Being aware of such complications is crucial in order to avoid them and treat them promptly should they arise.

## Introduction

Meniscal tears are the leading cause of knee injuries. Both medial and lateral menisci play a major role in knee joint stability as well as weight load dispersion. Meniscal preservation is crucial for joint longevity and joint degenerative disease prevention [1]. Multiple surgical modalities were implemented to confront such injuries. They can range from non-surgical treatment to total meniscectomies. Complete meniscectomies are no longer the mainstay of treatment due to their higher long-term risk of knee osteoarthritis [2]. Newer meniscal repair surgical techniques are being implemented, especially in younger athletic patients and in lesions located in the well-vascularized peripheral zones [3]. The arthroscopic approaches currently implemented include the outside-in, inside-out, and all-inside techniques [4]. The most commonly used technique is currently the inside-out suture; however, since its introduction in the early '90s, the all-inside repair technique has gained popularity. This was mainly due to its decreased surgical time and decreased risk of adverse events when compared to the other techniques [5]. However, several complications have been reported using the all-inside procedure. These include injuries to the surrounding arteries and nerves, meniscal cyst formation, subcutaneous implant migration, foreign body reaction, and aseptic synovitis [6,7]. Newer versions of the all-inside ultrahigh-molecular-weight polyethylene sutures have been developed over the years, which use a redefined knot-pulley mechanism, which has been shown to decrease the rate of the previously mentioned complications [8].

In this paper, we report a case of semimembranosus tendon entrapment following an all-inside ramp meniscal lesion repair. Since we are not aware of such an association, a detailed report of this clinical case would be relevant in clinical practice.

## Case presentation

**Figure 1:**
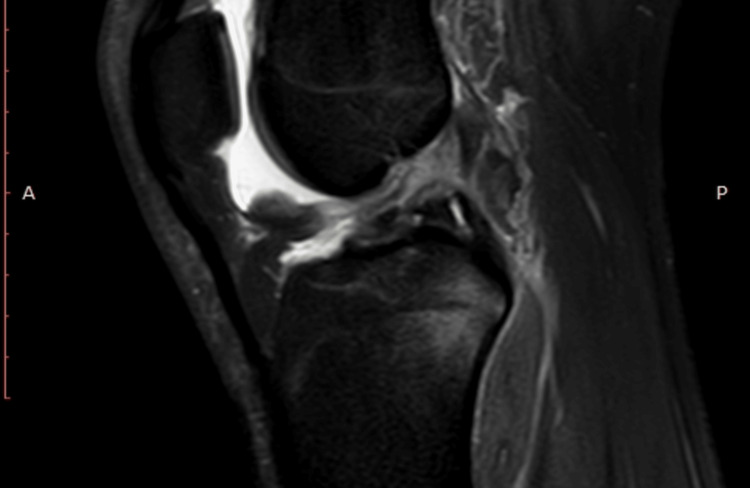
MRI showing anterior cruciate ligament (ACL) tear

**Figure 2:**
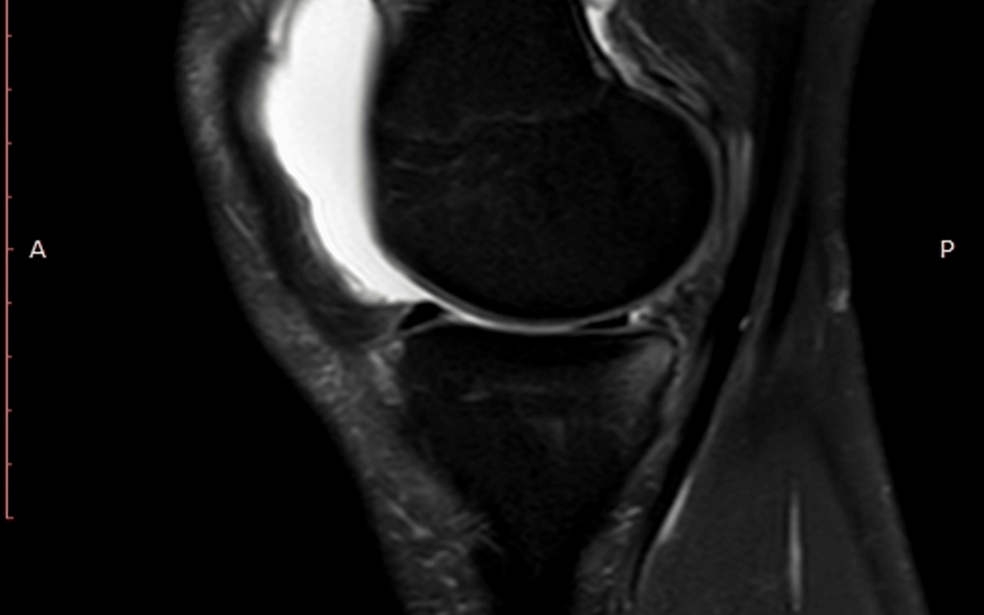
MRI showing ramp lesion

An 18-year-old male patient presented to our clinic with right medial compartment knee tenderness following a twisting injury during a soccer game. An evaluation revealed a complete anterior cruciate ligament (ACL) tear with an associated meniscal ramp lesion (Figures 1-2).

Arthroscopic ACL reconstruction was performed using a bone-tendon-bone (BTB) graft and the medial meniscus ramp lesion was repaired using two Mitek Truespan Meniscal Repair System (DePuy Synthes, Raynham, Massachusetts, United States) all-inside vertical mattress sutures.

No intra-operative or post-operative complications were noted. The conventional rehabilitation program was followed with partial weight bearing for three weeks post-op, followed by gradual full weight bearing up to six weeks post-op.

Upon his first follow-up visit three weeks post-op, the patient had regained knee mobility of 0-90°, stability of the knee (Lachman negative) without any tibial translation, and no complaints of pain at the joint line or on stress testing. At six weeks follow-up, the patient had achieved full mobility in both flexion and extension and had a physical examination similar to his first post-op visit. Rehabilitation protocol was continued as planned and a follow-up was scheduled at three months. Eight weeks following the surgery, the patient started to develop posterior knee pain upon full extension with loss of complete knee extension (5° lag). Physical examination revealed stability of the knee joint with no signs of meniscal injury (Mcmurray negative and no pain at the joint line). The patient reported point tenderness at the medial aspect of the posterior knee at the insertion of the hamstring muscles (semimembranosus and semitendinosus insertion). Intensive physical therapy and pain management were recommended with no further diagnostic imaging ordered and the patient was asked to follow up in two weeks.

**Figure 3:**
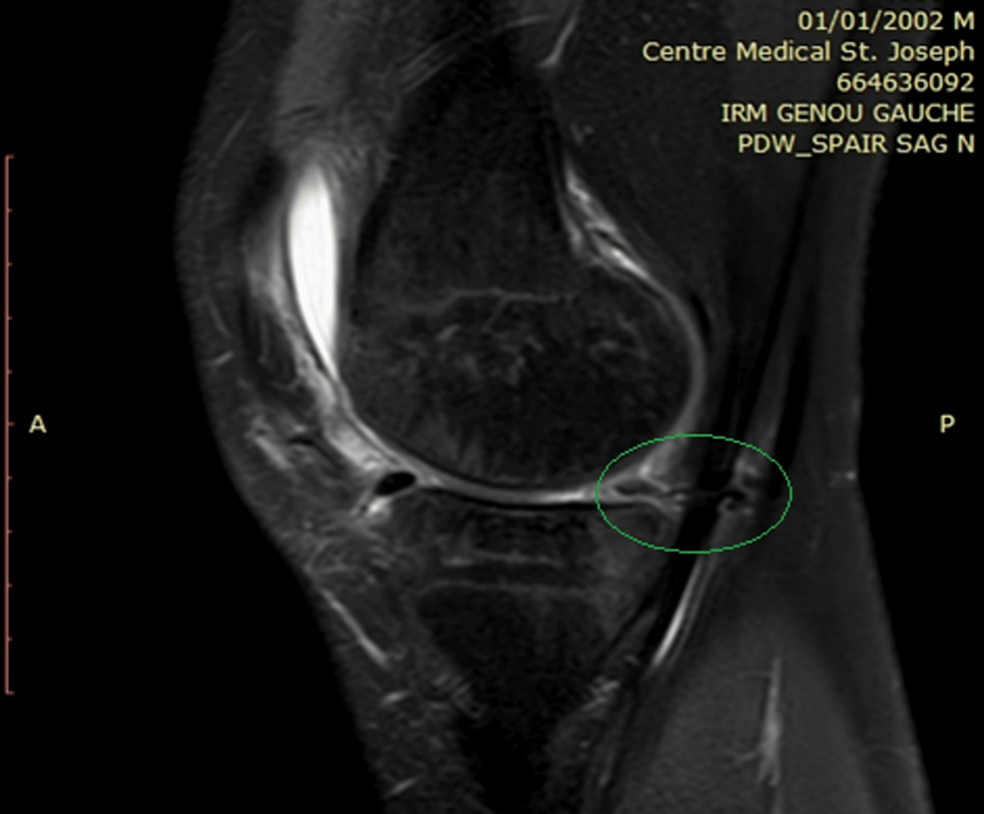
MRI of the left knee showing entrapment of semimembranosus tendon by medial meniscal suture

**Figure 4:**
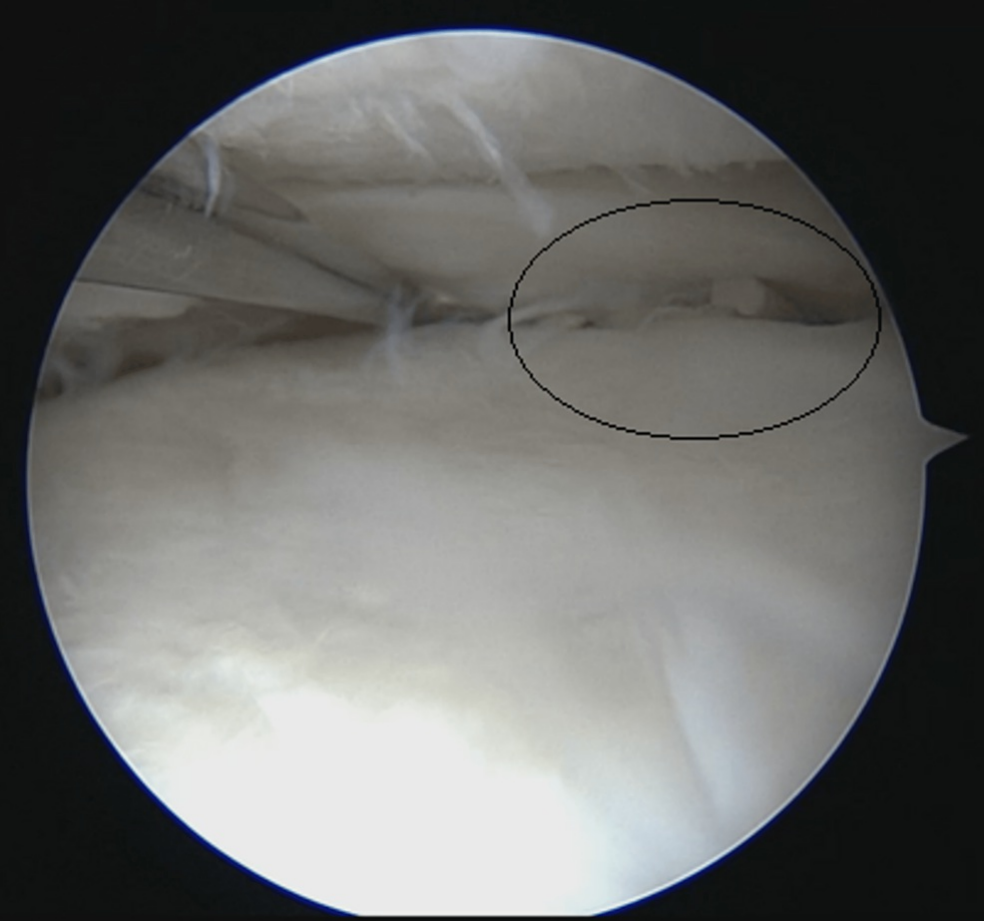
Medial meniscus suture and complete healing of medial meniscal lesion seen during left knee arthroscopy

During his fourth visit, the patient showed no signs of improvement with the persistence of posterior knee pain upon extension with a snapping sensation. An MRI of the knee (Figure 3) was performed that revealed semimembranosus tendon entrapment by the meniscal suture. Conservative management was attempted at first to allow healing of the meniscal tear and for the possibility of spontaneous release of the meniscal suture by hamstring pull. However, at three months post-operatively, failure to improve prompted a surgical intervention. Diagnostic knee arthroscopy, using the standard medial and lateral portals, was performed that revealed complete healing of the previous meniscal tear (Figure 4).

**Figure 5:**
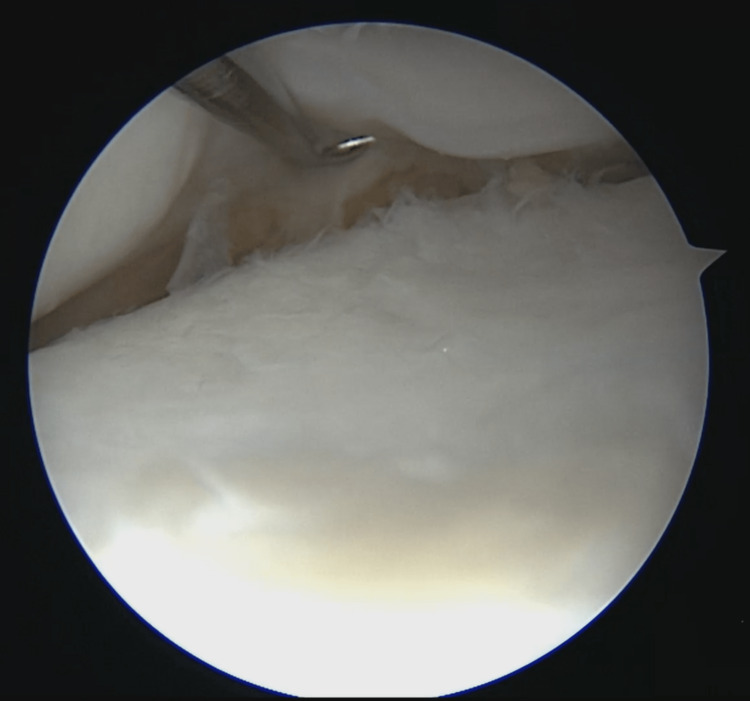
Left medial meniscus after releasing most medial suture showing complete healing

The medial all-inside meniscal sutures and plastic clips were identified, the most medial suture was released, and the plastic clips were removed, thus completely liberating the semimembranosus tendon (Figure 5). This was followed by a posteromedial approach and identification of both medial hamstrings to ensure that no plastic clips remained on the tendons.

Post-operatively, the patient was allowed full weight bearing and the patient reported immediate relief of his previous symptoms, and rehabilitation protocol was resumed normally.

## Discussion

When dealing with a meniscal injury, the surgical repair technique should be tailored to the patient’s demographics, type of tear, size, location, and associated ligamentous lesions. Understanding the knee joint anatomy in detail is crucial to prevent iatrogenic injuries to the structures around the menisci and to be able to identify them in case of complaints post-operatively; surgeons should be aware of all potential complications [9]. We report a case of a medial meniscal suture, using a Mitek Truespan anchoring to the semimembranosus tendon with symptomatic pain and knee extension lag at six weeks post-operatively. To our knowledge, there is no previous literature describing such complication after an all-inside technique.

Although the introduction of newer all-inside devices has made it easier and faster to repair torn menisci arthroscopically, the clinical outcomes of such surgeries are still surgeon and technique dependent [7]. With the Mitek Truespan device, the surgical technique involves adjusting the depth stop depending on the meniscal tear, which is usually set between 16-20 mm depending on the knee size; the device is inserted into the knee joint through the appropriate portal depending on the tear location. Then, the meniscal tissue is penetrated by the needle in the desired area to deploy the first implant after which the device is slightly retracted backward and re-inserted to deploy the second implant in an adjacent area, usually 6-9 mm away from the first one. The device is then fully retracted from the joint space and the pre-tied suture is pulled manually to the desired tension and cut at the meniscal surface. The same steps are repeated as needed to fully cover the tear. Entry point, device positioning, and depth of deployment are critical steps during this procedure and failure to perform them adequately can lead to major complications like repair failure, neurovascular compromise, and adjacent chondral and ligamentous damage [10].

**Figure 6:**
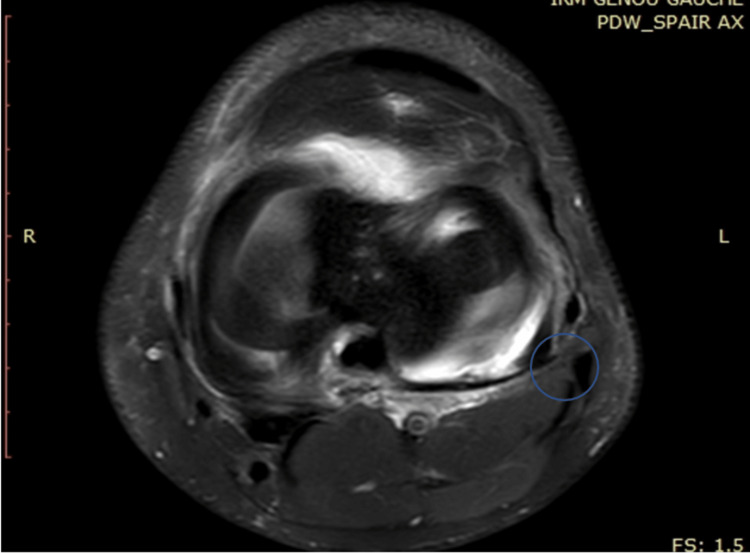
MRI showing the proximity of the semimembranosus tendon to the medial meniscus

Soft tissue entrapment including popliteal artery entrapment, sartorius tendon entrapment, and semimembranosus tendon entrapment have all been reported using the inside-out and outside-in suturing techniques [11] and these all could be considered differential diagnoses. To our knowledge, this case is the first report of semimembranosus tendon entrapment using the all-inside technique. The proximity of the semimembranosus tendon insertion can possibly explain the mechanism of entrapment in this case (Figure 6).

In fact, distal attachment of the semimembranosus tendon to the posterior horn of the medial meniscus is well described in the literature both anatomically and radiologically [12]. An incorrect depth of firing could have pushed the suture far beyond the posterior capsule, attaching it to the proximally located semimembranosus tendon. This, in turn, could have led to the symptoms seen in our patient. The hamstring muscles usually contract/shorten during flexion and relax/lengthen during extension of the knee. An unwanted suture connecting the medial meniscus to the semimembranosus tendon prevents its full relaxation during knee extension, thus inducing pain and inability to fully extend the knee. Surgical repair of this complication was needed to release the tendon, not only for symptomatic relief but also to prevent re-injury of the medial meniscus and/or tendon rupture with repeated activity.

## Conclusions

It is therefore crucial to be aware of such a complication when doing an all-inside suturing of a ramp meniscal lesion. It will help avoid its occurrence by doing a proper joint measurement, device positioning, and firing depth adjustment. Being aware of such a complication will also help to identify it and repair it early on whenever suspected, without further complications.

## References

[1] Barber FA, Howard MS, Ashraf W, Spenciner DB (2020). The biomechanical performance of the latest all-inside meniscal repair devices. Arthroscopy.

[2] Bogunovic L, Kruse LM, Haas AK, Huston LJ, Wright RW (2014). Outcome of all-inside second-generation meniscal repair: minimum five-year follow-up. J Bone Joint Surg Am.

[3] De Maeseneer M, Shahabpour M, Lenchik L, Milants A, De Ridder F, De Mey J, Cattrysse E (2014). Distal insertions of the semimembranosus tendon: MR imaging with anatomic correlation. Skeletal Radiol.

[4] Espejo-Baena A, Golano P, Meschian S, Garcia-Herrera JM, Serrano Jiménez JM (2007). Complications in medial meniscus suture: a cadaveric study. Knee Surg Sports Traumatol Arthrosc.

[5] Gliatis J, Kouzelis A, Panagopoulos A, Lambiris E (2005). Chondral injury due to migration of a Mitek RapidLoc meniscal repair implant after successful meniscal repair: a case report. Knee Surg Sports Traumatol Arthrosc.

[6] Gwathmey FW Jr, Golish SR, Diduch DR (2012). Complications in brief: meniscus repair. Clin Orthop Relat Res.

[7] Perdue PS, Hummer CD, Colosimo AJ, Heidt RS, Dormer SG (1996). Meniscal repair: Outcomes and clinical follow-up. Arthroscopy.

[8] Laurendon L, Neri T, Farizon F, Philippot R (2017). Prognostic factors for all-inside meniscal repair. A 87-case series. Orthop Traumatol Surg Res.

[9] Patil SS, Shekhar A, Tapasvi SR (2017). Meniscal preservation is important for the knee joint. Indian J Orthop.

[10] Petersen W, Karpinski K, Bierke S, Müller Rath R, Häner M (2022). A systematic review about long-term results after meniscus repair. Arch Orthop Trauma Surg.

[11] Turman KA, Diduch DR, Miller MD (2009). All-inside meniscal repair. Sports Health.

[12] Warth LC, Bollier MJ, Hoffman DF, Cummins JS, Hall MM (2016). New complication associated with all-inside meniscal repair device: ultrasound-aided diagnosis and operative localization of foreign body reaction. Orthop J Sports Med.

